# “Precovery” versus recovery: Understanding the role of cherry juice in exercise recovery

**DOI:** 10.1111/sms.14141

**Published:** 2022-02-14

**Authors:** Malachy P. McHugh

**Affiliations:** ^1^ Nicholas Institute of Sports Medicine and Athletic Trauma Lenox Hill Hospital at MEETH New York New York USA; ^2^ Faculty of Health and Life Sciences Northumbria University Newcastle UK

**Keywords:** antioxidant, inflammation, muscle function, nutrition, tart cherries

## Abstract

Cherry juice has become a standard component of athlete recovery strategies. This review covers the history of cherry juice as a recovery drink to give context to its current use. Fifteen studies were identified that included a measure of muscle function, soreness, or inflammation on the days following exercise and had an exercise insult sufficient to assess the effectiveness of the tart cherry intervention. Eight studies used a concentrated juice, three used a juice from fresh‐frozen cherries, two used a tart cherry concentrate gel, and two used a tart cherry powder. The effective juice dose was specific to the type of drink (fresh‐frozen versus concentrate) but dose‐response studies are lacking, and thus, the optimal dose for any specific type of cherry juice is not known. Timing of the dosing regimen is a critical factor. Studies have uniformly shown that muscle function will recover faster on the days after exercise if juice is provided for several days prior to exercise. Effects on soreness or systemic inflammation are more equivocal. The available evidence does not support a regimen that begins on the day of exercise or post‐exercise. Tart cherry powder did not enhance any metric of recovery on the days after exercise. In conclusion, the term recovery implies an intervention that is introduced after an exercise insult. The term “precovery” may be preferable to describe interventions that should be introduced on the days prior to exercise to facilitate recovery on the days after exercise. The evidence supports cherry juice as a precovery intervention across a range of athletic activities.

## 1 RATIONAL FOR REVIEW

Cherry juice has been extensively studied for its benefits for exercise recovery (for review, see Hill et al^1^) and has become a standard component of athlete recovery strategies. In a recent consensus statement on nutrition in elite football, there was a brief mention of the role of cherry juice in a section on recovery from match play.^2^ It was noted that cherry juice has become a popular recovery intervention, but it was shown not to be effective in football, with one study cited.^3^ The consensus conclusion was that “the available evidence does not support its specific use in football.” This interpretation of the Abbott et al. study^3^ and the overall conclusion highlights a misconception of the role of cherry juice in exercise recovery. The athletes in the study by Abbott et al.^3^ consumed a tart cherry concentrate gel on the day of the exercise and the subsequent days. Typically, cherry juice is consumed for several days prior to the exercise insult, in addition to the day of exercise and the subsequent days. The problem arises in the interpretation of the term recovery as something one does after an event. In this regard, cherry juice should not be regarded as a recovery drink, and it should be regarded as a “precovery” drink, where the term precovery implies an intervention prior to an athletic event.

The purpose of this review is to provide some historical context for the science behind cherry juice as a recovery intervention in sports and exercise. Understanding the genesis of the dosing regimens employed in the early research will provide a better understanding of the disparate subsequent research. While systematic reviews and meta‐analyses emphasize the application of strict processes and procedures to assimilate disparate studies, this narrative review will emphasize the nuances that explain conflicting findings. The goal is to provide a practical understanding of the role of cherry juice in exercise recovery. The content is specific to situations where the goal is recovery of function to maintain performance and does not address situations where the goal is to optimize training adaptations, in which case a recovery intervention may not be indicated.

## 2 THE GENESIS OF CHERRY JUICE AS A RECOVERY DRINK

In 2003, Jacob et al.^4^ showed that consumption of a bowl of sweet cherries (280 g, approximately 45 cherries) acutely lowered plasma urate and increased indices of antioxidant capacity in healthy women. A subsequent study^5^ included men and women and extended the intervention to 280 g of sweet cherries daily for 28 days. The notable new finding was that indices of inflammation were reduced, and specifically, C‐reactive protein was reduced by 25% and nitric oxide production was reduced by 18%. There was no effect on interleukin‐6 or tumor necrosis factor alpha. Based on the anti‐inflammatory and antioxidant effects seen with eating a bowl of 45 sweet cherries daily for 28 days, Connolly et al.^6^ examined whether consumption of a cherry juice was effective at reducing indices of exercise‐induced muscle damage. This was the first study in humans showing cherry juice to be effective in exercise recovery. Two 355 ml servings (2x12 fl oz) of cherry juice were given daily for 3 days prior to exercise, on the day of exercise, and on the subsequent 4 days. The drink was made using fresh‐frozen tart cherries. There were approximately 50–60 cherries in each serving. This dosing regimen was based on the findings of Kelley et al.^5^ showing that eating 45 sweet cherries a day had systemic antioxidant and anti‐inflammatory effects. It was presumed that there would be degradation of the phytonutrients during processing of a drink and that the end product would not have the potency of 50–60 fresh cherries. Therefore, two 35 ml servings were given each day to achieve a dose that was more than twice the number of cherries given in the prior studies.^4^, ^5^ Subsequently, the processing of this particular cherry juice drink was refined such that approximately 50–60 cherries could be provided in a 237 ml serving (8 fl oz). A dosing regimen of two 237 ml servings a day was replicated in several subsequent studies, all of which demonstrated efficacy across diverse conditions.^7^, ^8^, ^9^, ^10^

## 3 WHY TART CHERRY JUICE AND NOT SWEET CHERRY JUICE?

Both tart cherries and sweet cherries have been shown to have health benefits when consumed in sufficient amounts (for review, see Kelley et al^11^). While the phenolic concentration and composition vary between sweet and tart cherries, they also vary between different cultivars of tart or sweet cherries.^11^ Generally, both tart and sweet cherries have a range of different phytonutrients that have both antioxidant and anti‐inflammatory effects.^11^ In the original studies indicating potential health benefits from cherries, subjects ate bowls of Bing sweet cherries.^4^, ^5^ In the subsequent studies, on exercise recovery subjects drank commercially available Montmorency tart cherry juice.^2^, ^6^, ^8^, ^12^, ^13^, ^14^, ^15^, ^16^, ^17^, ^18^, ^19^, ^20^, ^21^, ^22^, ^23^, ^24^ The fact that commercial juices are made from tart cherries and not sweet cherries is a matter of cost and availability rather than differences in the phenolic concentrations between them. Pitting and juicing 50–100 sweet cherries are a viable alternative to purchasing a ready‐made tart juice, but this would be costly, time‐consuming, and dependent on the seasonal availability of cherries.

The exclusive use of Montmorency cherries in the exercise recovery studies does not mean that other cultivars are not as effective. Montmorency cherries are grown predominantly in Michigan in the United States. However, Eastern Europe is one of the largest cherry‐growing regions in the world (Turkey, Ukraine, and Poland) with different cultivars of sweet and tart cherries predominating depending on the specific geographic location. To date, there has been no research on exercise recovery examining the potential benefits of cherry juice from these regions and there has only been limited work on the differences in phenolic contents between different cultivars.

## 4 COMPARISONS OF PHENOLIC CONTENTS OF PRODUCTS USED IN EXERCISE RECOVERY STUDIES

A total of 19 studies have tested the effectiveness of six different tart cherry products for improving exercise recovery in humans. Four of the 19 studies tested juices made from fresh‐frozen cherries,^6^, ^8^, ^13^, ^19^ of which three were the same product.^6^, ^8^, ^13^ The juices used in these studies were reported to have a total phenolic content of at least 600 mg and an anthocyanin content of at least 40 mg. Ten of the 19 studies used a juice made from concentrate (all 10 used the same product).^14^, ^15^, ^16^, ^17^, ^18^, ^20^, ^21^, ^22^, ^23^, ^24^ The most recent of these studies^24^ reported a total phenolic content of 20.2 mg/ml and an anthocyanin content of 7.2 mg/ml. This amounts to 605 mg and 216 mg per 30 ml serving for total phenolic content and anthocyanin content, respectively. The earliest of the studies using this juice^17^ reported an anthocyanin content of 9.1 mg/ml (273 mg per 30 ml serving) but did not report a total phenolic content. Three of the 19 studies used a tart cherry powder, with two reporting a phenolic content of 991 mg and an anthocyanin content of 66 mg per serving.^25^, ^26^ The other reported 773 mg for phenolic content and 64 mg for anthocyanin content.^27^ The remaining two studies used a tart cherry concentrate gel^3^, ^28^ but did not report the phenolic or anthocyanin content. In one of these studies, the gel was diluted to replicate the placebo drink.^3^ The gel products are essentially a concentrate with a gel agent added to increase viscosity. Therefore, one might assume that the gel products would have a similar phenolic content to the juice concentrates.

It is unclear how the reported anthocyanin content for the cherry juice concentrate used in most studies is 3.3–6.8 times higher than for either the tart cherry powder or the juice from fresh‐frozen cherries. The skins of Montmorency cherries contain most of the anthocyanins, and the tart cherry powder used in two studies^25^, ^26^ was exclusively derived from the skins. The total phenolic content per serving was comparable between the juice from fresh‐frozen cherries and the juice concentrate, with 28%–65% higher values for the tart cherry powder.

Besides the type of juice (fresh‐frozen versus concentrate versus powder), the 19 studies differed in exercise mode, study population, dosing regimen, and the number and type of outcome measures. One issue with regard to potential health‐related or exercise recovery benefits comparing a juice concentrate versus a juice using fresh‐frozen cherries is that harsher processing techniques are used in making a concentrate. Degradation of the phytonutrients during the processing is unavoidable and will be greater in the production of a concentrate. In two separate studies, anthocyanin content was shown to be 60%^29^ and 57%^30^ lower in Montmorency cherry concentrate versus fresh‐frozen Montmorency cherries. In one of these studies, antioxidant activity was 65% lower in concentrate versus fresh‐frozen cherries.^29^ However, in the other study the opposite was the case, and antioxidant activity was 60% lower per serving in the fresh‐frozen versus concentrate.^30^ Additionally, in that study anti‐inflammatory activity was also lower in fresh‐frozen versus cherry concentrate.^30^ The limited and conflicting research in the area makes it difficult to make practical conclusions on potential differences in health or recovery benefits of cherry juice from fresh‐frozen cherries versus cherry concentrate.

An additional consideration, regardless of the type of drink, is that post‐production storage affects degradation of the phytonutrients, with heat, and exposure to sunlight, decreasing the effective shelf life. Thus, refrigeration will be advantageous for maintaining potency of any particular cherry juice. To some extent, the recommended dosing regimens for commercially available concentrate versus fresh‐frozen juices attempt to account for the potential differences in drink potency with the estimated number of cherries per serving substantially higher in the concentrate (see section 5.1 and 6.3).

## 5 DOSE AND BIOAVAILABILITY

### 5.1 Dosing regimens in exercise recovery studies

The cherry juice dosing regimens employed in the various studies on exercise recovery have been specific to the actual product being studied. A regimen of two servings a day (355 ml^6^ or 237 ml^8^, ^13^) for several days before exercise and for a couple of days after exercise has been employed in studies using a cherry juice made from fresh‐frozen Montmorency tart cherries. In these studies, it was estimated that participants were taking the equivalent of approximately 100 cherries per day. A regimen of two 30 ml servings a day has been employed in exercise recovery studies using a Montmorency cherry juice concentrate.^14^, ^15^, ^16^, ^17^, ^18^, ^20^, ^21^, ^22^, ^23^, ^24^ In these studies, it was estimated that participants were taking the equivalent of approximately 180 cherries per day.

### 5.2 Dose‐response studies

Four of the exercise recovery studies measured total antioxidant status after the pre‐exercise dosing period.^8^, ^17^, ^25^, ^26^ In one study, a regimen of two 474 ml a day of cherry juice from fresh‐frozen cherries for 4 days was shown to increase total antioxidant status by 11%.^8^ By contrast, total antioxidant status was not different from control after 6 days of 60 ml tart cherry juice concentrate per day^17^ or after seven daily ingestions of 480 mg powdered tart cherry capsules.^25^, ^26^ In a non‐exercise study, a dosing regimen of 30 ml tart cherry juice concentrate per day for 42 days resulted in a 7% increase in antioxidant status.^31^ Taken together, these five studies^8^, ^17^, ^25^, ^26^, ^31^ indicate that cherry juice from fresh‐frozen cherries^8^ may more readily affect antioxidant status than juice from cherry concentrate^17^ or a tart cherry powder^25^, ^26^ when the dosing period is 5–7 days. Extending the period of consumption of the cherry juice concentrate to seven weeks can increase antioxidant status. However, an increase in antioxidant status may not be the primary mechanism for improved recovery since indices of recovery were affected despite no change in pre‐exercise antioxidant status.^17^ Cherries have been shown to have a potent anti‐inflammatory effect by inhibiting cyclooxygenase enzyme activity.^30^, ^32^ This effect was shown to be superior to the effect of aspirin but inferior to ibuprofen.^32^

One study compared the effects of different doses of the same cherry juice.^33^ There were no differences in the responses to a dosing regimen of 30 ml (approximately 90 cherries) versus 60 ml (approximately 180 cherries) of cherry juice concentrate per day for 2 days. Both doses acutely reduced systemic inflammation after the first serving, but values had returned to baseline the next day. Repeating the dose on the second day did result in a sustained reduction in systemic inflammation, with C‐reactive protein (CRP) values approximately 35% below baseline on the third day, with similar effects for each dose. Thus, tart cherry juice concentrate can have systemic effects that could be beneficial for exercise recovery, but it appears to take several days to achieve a sustained effect. By comparison, eating a bowl of sweet cherries each day resulted in a non‐significant 8% decline in CRP after 8 days and a significant 25% decline after 28 days.^5^ Since healthy men and women generally have extremely low CRP, it is not appropriate to gauge the effectiveness of cherry juice dosing regimens simply on changes in baseline CRP. Testing the effectiveness of cherry juice dosing regimens on CRP in non‐exercise studies requires populations with elevated CRP. In patients with mild to moderate arthritis, CRP was higher than normal and a dosing regimen of 474 ml per day of cherry juice from fresh‐frozen cherries for six weeks resulted in a 23% reduction in CRP.^10^

In a prior review of the health benefits of cherry juice, Bell et al.^34^ acknowledged that it was unclear whether the exercise recovery benefits were due to pre‐exercise consumption, post‐exercise consumption, or the combination of both. To date, no study has formally examined this issue but based on changes in antioxidant status^8^, ^17^, ^25^, ^26^ and systemic inflammation^5^, ^10^, ^33^ a pre‐exercise dosing period would seem to be needed.

**TABLE 1:** Dosing regimens for exercise recovery studies

Reference	Type of juice	Mode of exercise	Sample Size	Daily dose (#cherries)	#Days Preexercise	#Days Post‐exercise	Total dose	Pre‐exercise dose	Indices of recovery
Bell et al^15^	Concentrate^a^	Cycling	8	180	4	3	1260	720	CRP +others
Bell et al^16^	Concentrate^a^	Cycling	8	180	4	3	1440	720	MVC, soreness, CRP +others
Bell et al^14^	Concentrate^a^	Simulated Soccer	8	180	4	3	1440	720	MVC, CMJ, soreness, CRP +others
Bowtell et al^17^	Concentrate^a^	Isotonic Quadriceps	10	180	6	2	1620	1080	MVC, soreness, CRP +others
Brown et al^18^	Concentrate^a^	Intermittent Sprinting	10	180	4	3	1440	720	MVC, CMJ, soreness, CRP +others
Lamb et al^20^	Concentrate^a^	Eccentric Elbow Flexors	12	180	4	4	1620	720	MVC, soreness +others
Quinlan and Hill^23^	Concentrate^a^	Simulated Soccer	10	180	5	2	1440	900	MVC, CMJ, Soreness, CRP +others
Wangdi et al^24^	Concentrate^a^	Eccentric Quadriceps	10	180	7	2	1800	1260	MVC, CMJ, Soreness, CRP +others
Abbott et al^3^	Concentrate (gel)^b^	Soccer Match	10	200	0	2	600	0	CMJ, Soreness +others
Kupusarevic et al^28^	Concentrate (gel)^b^	Rugby Match	10	200	2	2	1000	400	Soreness
Connolly et al^6^	Fresh‐frozen^c^	Eccentric Elbow Flexors	14	110	3	4	880	330	MVC, soreness +others
Hillman et al^13^	Fresh‐frozen^c^	Plyometric Exercise	8	90	5	4	900	450	CMJ, soreness +others
Howatson et al^8^	Fresh‐frozen^c^	Marathon Run	10	110	5	2	880	550	MVC, soreness, CRP +others
Beal et al^27^	Powder^d^	Eccentric Quadriceps	16	N/A	4	7	N/A	N/A	MVC, soreness +others
Lever et al^25^	Powder^e^	Squatting	11	N/A	7	2	N/A	N/A	MVC, soreness +others

Note

Sample size refers to cherry treatment. Pre‐exercise does not include any juice consumed on the day of exercise. N/A=information was unavailable on number of cherries in a serving. Product information: ^a^CherryActive, ^b^Healthspan Ltd, ^c^CherryPharm/Cheribundi, ^d^TartVitaCherry, and ^e^CherryPURE.

Abbrevaitions: CMJ, Countermovement jump; CRP, C‐reactive protein; MVC, Maximal voluntary contraction.

### 5.3 Bioavailability studies

Data on the bioavailability of the phytonutrients in cherries are very limited. Kirakosyan et al. demonstrated a diverse distribution of tart cherry anthocyanins across different tissues after supplementing rats’ diets with tart cherry powder for seven weeks.^35^ Anthocyanin content was highest in the bladder but also evident in liver, kidney, and brain tissue. Unfortunately, there was no measurement of anthocyanin content in muscle. One study in humans showed that plasma levels of phenolic acids increased by 2–3 times baseline within 1–2 h of consuming 30 ml or 60 ml of tart cherry juice concentrate.^36^ However, plasma levels were mostly back to baseline by eight hours, indicating a transient acute effect. In a more recent study,^24^ consuming 30 ml of tart cherry juice concentrate twice daily for seven days resulted in elevations in plasma levels of some phenolic acids compared to placebo. More importantly, this study^24^ showed increased expression of antioxidant genes and proteins in skeletal muscle after seven days of cherry juice consumption. Cherry juice consumption also enhanced recovery from eccentric exercise‐induced muscle damage (see section 6.4 and Table 2).

## 6 COMPARISON OF THE EXERCISE RECOVERY BENEFITS BETWEEN CHERRY JUICE STUDIES

### 6.1 Inclusion criteria for exercise recovery studies

The literature on the exercise recovery benefits of cherry juice includes studies with a range of different exercise modes, using different types of cherry juice, with different dosing regimens and different indices of recovery. In order to assimilate the findings, studies were selected based on four criteria:

1. The study was a randomized trial on humans.
2. The study included a placebo control.
3. Recovery indices included at least one of the following: (1) an assessment of strength using a maximal voluntary contraction (MVC) or jump performance using a countermovement jump (CMJ), (2) an assessment of soreness, and (3) a measurement of CRP (an index of systemic inflammation).
4. The study involved an exercise intervention sufficient to impair muscle function, cause soreness, or increase systemic inflammation in the control condition.
5. The study included measurements of recovery one and two days after the exercise.

Fifteen studies met the inclusion criteria (Table 1). Eight studies used tart cherry juice concentrate, two studies used tart cherry concentrate gel, three used juice from fresh‐frozen tart cherries, and two used a tart cherry powder, one served as a diluted drink^27^ and one in a capsule.^25^ The number of cherries per serving and the number of servings are reported, with differentiation according pre‐ versus post‐exercise supplementation (Table 1). The cherries in all 15 studies were Montmorency tart cherries.

Four studies were excluded.^19^, ^21^, ^22^, ^26^ In a study of recovery after a rugby match, the game did not induce sufficient impairments to assess the potential benefit of a recovery intervention.^22^ For example, CMJ was assessed one day prior to and two days after the game. Decrements in flight time were only 3.8% in the control condition and 3.7% in the treatment condition. It would have been preferable to have measurements one day after exercise when there would have been a greater impairment in CMJ and, therefore, greater potential to detect an effect of a recovery intervention. Additionally, this was a crossover study with juice or placebo provided for five days prior to the match, on the day of the match, and for two days after the match. The study was carried out over two consecutive weeks, with games on consecutive Saturdays. That left only four days for switching to opposite treatment before the second game since participants continued the initial condition for two days post‐game. Thus, the dosing regimen could not have been replicated for each condition. More importantly, the lack of a washout period between treatments is a major confounding factor. One similar study using a crossover used a 6‐day washout period.^6^ A second study that was excluded involved water polo players in a crossover design.^21^ Supplementation with cherry juice or placebo began at the start of a 6‐day training regimen, with a match simulation on day 6, and a 5‐week washout period between sessions. Four performance measures were made prior to the first training session and again the day after the match simulation on day 6. However, none of the four performance measures showed a statistically significant decrement for either condition; thus, it was not possible to assess the potential benefits of a recovery intervention. A third study was excluded because the only outcome measure was soreness, and it was only recorded at the end of a 24‐h relay run and not recorded on subsequent days.^19^ The athletes consumed a juice from fresh‐frozen tart cherries twice daily for 7 days before the race and on the day of the race. Post‐race soreness was significantly lower in the cherry juice group (22.6 ± 12.6 vs. 45.3 ± 20.5 on a 100‐point scale). A final study that was excluded compared tart cherry powder capsules to placebo taken for seven days prior to a half marathon race and on 2 days after the race.^26^ Multiple markers of recovery were recorded, including markers of soreness, inflammation, oxidative stress, and hormonal stress. The inflammatory markers included numerous interleukins and other inflammatory markers but did not include a CRP measure. The primary limitation in assessing the recovery benefits of the intervention was that, prior to the run, the subjects already had significant soreness, and more importantly, the soreness was greater in the placebo group versus the tart cherry group. Thus, it was not possible to assess the effect of the intervention of soreness. There was no measure of muscle function. While there were some statistically significant effects of the intervention on a few markers in the immediate post‐exercise period, no markers differed between tart cherry and placebo 24 and 48 h after the exercise.

### 6.2 Method of comparison between exercise recovery

For each of the 15 studies reporting changes in MVC, CMJ, soreness, or CRP at 24 and 48 hr after exercise, an index of protection was calculated for the effects of cherry juice (Table 2). For these calculations, the changes in the control condition were compared to the changes in the cherry juice condition. For example, if MVC was 70% of baseline one day post‐exercise in the control condition and 85% of baseline in the cherry juice condition, the index of protection on that day would be 50% (15% change from baseline in cherry juice condition divided by 30% change from baseline in control of condition). If in the original study there was a non‐significant change in the control condition for a given marker, an index of protection was not calculated because the exercise stimulus was insufficient to test the efficacy of cherry juice. If the change from baseline in the cherry juice condition was better than baseline (e.g., improved strength), the index of protection was recorded as 100%. If the change from baseline was worse in the cherry juice condition versus control, the index of protection was recorded as 0%. If an index of protection was calculated for a non‐significant change, it is indicated in the table by NS after the protection value.

Of the 19 exercise recovery studies that are cited for testing a cherry product (15 meeting the inclusion criteria), seven used a crossover design such that all subjects received the experimental and placebo treatments.^3^, ^6^, ^17^, ^21^, ^22^, ^24^, ^28^ Besides the need for an adequate washout period between treatments, a crossover design introduces the potential confounding effect of the repeated bout effect. Three of the seven crossover studies^6^, ^17^, ^24^ used a contralateral limb design, whereby the exercise insult was applied to the contralateral limb after the second treatment. This diminishes, but does not eliminate, the confounding effect of the repeated bout effect. The other four crossover studies^3^, ^21^, ^22^, ^28^ involved team sports. For three of these studies,^3^, ^22^, ^28^ the exercise was match play, so the athletes should have been sufficiently exposed to the stress that any repeated bout effect would be small or absent. The other involved training exercises and simulated play^21^ and might have incurred a repeated bout effect. However, the exercise stimulus was insufficient to affect the recovery metrics and the effectiveness of the intervention could not be assessed.

### 6.3 Summary of dosing regimens and exercise interventions

(Table 1) Of the 15 studies that met the inclusion criteria, the exercise stimulus was an actual or simulated field sport in five studies, endurance cycling in two studies, endurance running in one study, plyometric exercise in one study, and isolated eccentric or isotonic exercise of single muscle groups in five studies and multiple muscle groups in one study. The sample sizes ranged from eight to 16 with an average of 10 subjects per study. The average ( ± SD) number of days juice was consumed prior to exercise was 4.3 ± 1.8 with a range of 0 to seven days. Only one study did not provide juice on days prior to the exercise stimulus, providing juice on the day of exercise and on the subsequent two days.^3^ The total estimated dose in terms of number of cherries averaged 1508 ± 165 (range 1260 to 1800) for the studies using a juice made from concentrate, 540 and 1000 for the two studies using a gel, and 887 ± 12 for studies using a juice made from fresh‐frozen cherries (two had a total of 900 and the other 880 cherries). An estimate of total dose of cherries was not available for the two studies using a tart cherry powder. The total estimated dose of cherries provided on the days prior to exercise was 855 ± 210 (range 720–1260) for the studies using a juice made from concentrate, 0 and 200 for the two studies using a gel, and 443 ± 110 for the three studies using a juice made from fresh‐frozen cherries (330–550).

### 6.4 Summary of the index of protection provided by cherry juice

(Table 2) Eleven of the 15 exercise recovery studies that met the inclusion criteria assessed MVC, six assessed CMJ, four assessed MVC and CMJ, 13 assessed soreness, and eight measured CRP. Thirteen of the 15 studies had some measure of muscle function (MVC and/or CMJ); however, two of these had an insufficient exercise insult to affect either MVC or CMJ. Of the remaining 11 studies, eight showed enhanced recovery of function (MVC or CMJ); these included 6 of 7 studies using a cherry juice concentrate (4–7 days pre‐exercise dosing) and 2 of 2 studies using juice from fresh‐frozen cherries (3 days and 5 days pre‐exercise dosing). Of the three studies with no effect on function, one used a cherry juice concentrate (4 days pre‐exercise dosing), one used a cherry concentrate gel (0 days pre‐exercise dosing), and one used a tart cherry powder (7 days pre‐exercise dosing).

**TABLE 2:** Index of protection for indices of recovery

Reference	Index of protection MVC at 24 h	Index of protection MVC at 48 h	Index of protection CMJ at 24 h	Index of protection CMJ at 48 h		Index of protection Soreness at 24 h	Index of protection Soreness at 48 h	Index of protection CRP at 24 h	Index of protection CRP at 48 h
Bell et al^15^	Not tested		Not tested			Not tested		48%	42%
Bell et al^16^	100%	92%	Not tested			23%NS	39%NS	Exercise stimulus insufficient	
Bell et al^14^	100%	100%	42%	55%		34%	49%	27%NS	34%NS
Bowtell et al^17^	40%	37%	Not tested			27%NS	36%NS	Exercise stimulus insufficient	
Brown et al^18^	13%NS	57%NS	50%	67%		36%NS	19%NS	50%NS	80%NS
Lamb et al^20^	5%NS	7%NS	Not tested			0%	0%	Not tested	
Quinlan and Hill^23^	70%	86%	76%	100%		44%	74%	Exercise stimulus insufficient	
Wangdi et al^24^	38%	29%	0%	0%		37%NS	16%NS	Exercise stimulus insufficient	
Abbott et al^3^	Not tested		0%			0%	0%	Not tested	
Kupusarevic et al^28^	Not tested		Not tested			30%NS	51%NS	Not tested	
Connolly et al^6^	60%	74%	Not tested			15%	33%	Not tested	
Hillman et al^13^	Not tested		Exercise stimulus insufficient			37%NS	68%NS	Not tested	
Howatson et al^8^	45%	100%	Not tested		0%		0%	35%	33%
Beal et al^27^	Exercise stimulus insufficient		Not tested			0%	0%	Not tested	
Lever et al^25^	10%NS%	0%NS%	Not tested			51%NS	39%NS	Not tested	

Note

Values showing statistically significant improved recovery with cherry juice are in bold.

Abbreviations: CMJ, Countermovement jump; CRP, C‐reactive protein; MVC, Maximal voluntary contraction.

For the 10 studies that assessed MVC and had an adequate exercise stimulus, the average index of protection was 34 ± 30% one day after exercise and 58 ± 38% at two days. For the five studies that assessed CMJ and had an adequate exercise stimulus, the average index of protection was 48 ± 35% one day after exercise and 44 ± 39% at two days. One of the studies showed no protection used a single limb CMJ.^24^ The exercise was unilateral eccentric quadriceps exercise. Of note, cherry juice did protect against strength loss in that study.^24^

Soreness was recorded in 14 of the 15 studies. Cherry juice was effective at reducing soreness in three of the studies (index of protection 15%, 34%, 44% at 1 day, 33%, 49%, and 74% at 2 days). Of the 10 studies that showed no significant protection against soreness, four had zero protection, with the remaining six studies showing non‐significant protection ranging from 23% to 51% one day post‐exercise and from 19% to 68% two days post‐exercise. For all 14 studies recording soreness, the index of protection was 29 ± 18% one day post‐exercise and 30 ± 25% two days post‐exercise. There were no consistent distinctions in dose, timing, or exercise mode between the three studies showing protection against soreness and the 10 studies showing no protection.

CRP was measured in eight of the 15 studies, with two showing a significant protective effect of cherry juice (48% and 35% protection one day post‐exercise, and 42% and 33% protection two days post‐exercise), two showing non‐significant effects (27% and 50% one day post‐exercise, and 34% and 80% two days post‐exercise), and four having an insufficient exercise stimulus to affect CRP. The primary distinction between the studies showing a protective effect and those not showing a protective effect was the mode of exercise. Both studies with a protective effect involved endurance exercise (cycling and running), while both studies showing no protective effect involved simulated soccer activity (intermittent sprinting). The index of protection for the four studies with a sufficient exercise stimulus was 40 ± 11% on day 1 and 47 ± 22% on day 2. It is important to appreciate that the CRP values are small unless the exercise insult involves a large metabolic stress over a prolonged time, for example, running a marathon. Thus, the index of protection of 35% on day 1 and 33% on day 2 following a marathon^8^ are statistically significant and of clinical relevance, whereas the larger values for the index of protection for CRP that did not reach statistical significance indicate exercise insults that did not result in large CRP values.

## 7 CONCLUSIONS

### 7.1 Type of cherry juice or cherry product

Studies using cherry juice from concentrate or juice from fresh‐frozen cherries have consistently provided enhancement of recovery (at least one positive effect noted in 9 of 11 studies). No enhancements of recovery on the days after exercise were found for two studies using tart cherry concentrate gel or in two studies using a tart cherry powder (Tables 1 and 2). All of these studies used Montmorency cherries. No recovery studies have tested other tart cherry cultivars or sweet cherries.

### 7.2 Phenolic content of the cherry juice or product

The reported phenolic content of the cherry products appears to be unrelated to the subsequent effects on indices of recovery. Total phenolic content was highest for the tart cherry powder,^25^, ^26^, ^27^ but none of these studies showed effectiveness for enhancing recovery on the days after exercise. One of the studies^25^ showed cortisol levels to be lower in the tart cherry group versus control at 24 and 48 hours post‐exercise, but there were no differences between groups in strength, soreness, inflammation, or blood markers of muscle damage.

### 7.3 Timing of the dose: pre‐exercise dosing

Consuming cherry juice on the days prior to an exercise insult clearly protects muscle function across a range of types of physical exercise. Effects on soreness and systemic inflammation were more equivocal. Of the two studies that failed to show protection for a measure of muscle function, one used a tart cherry concentrate gel, starting supplementation on the day of the exercise insult, while the other used a 7‐day pre‐exercise supplementation with a tart cherry powder. The two studies with less than three days of pre‐exercise tart cherry consumption were both negative for all recovery metrics (both used a cherry concentrate gel). While the available data support pre‐loading for several days prior to exercise, no conclusion can be reached on the possible additional benefit of continuing cherry juice consumption on the subsequent days. All studies have continued consumption through the recovery period. In reality, for athletes trying to facilitate recovery between games over the course of a season, the best strategy would be to maintain cherry juice consumption during the entire season. This recommendation applies to sports and physical activities, where the schedule does not facilitate adequate recovery time between exposures.

### 7.4 Concentrate versus fresh‐frozen juice

While no study has directly compared a cherry juice concentrate to a juice from fresh‐frozen cherries, there are two very comparable studies that provide some insight.^6^, ^20^ Connolly et al.^6^ used a 3‐day pre‐exercise dose, continuing the day of exercise and for the next four days using a juice from fresh‐frozen cherries. Lamb et al.^20^ used a 4‐day pre‐exercise dose, continuing the day of exercise and for the next 3 days with a juice from concentrate. Both studies used eccentric elbow flexor exercise to induce damage; Lamb et al. used 50 eccentric isokinetic MVCs, and Connolly et al. used 40 eccentric isotonic MVCs. Connolly et al. showed beneficial effects for recovery of strength and soreness, while Lamb et al. showed no effects on strength or soreness. The strength loss in the control condition was comparable between studies 30% (Connolly et al) vs. 25% (Lamb et al) on day 1 and 27% vs. 22% on day 2. This indicates that the exercise insults were similar between studies. Strength loss in the cherry juice condition was 12% vs. 24% and 7% vs. 21%, respectively. These findings are consistent with the literature indicating that juice from fresh‐frozen tart cherries more readily affects total antioxidant status than juice from concentrate or tart cherry powder (see section 5.2).

### 7.5 Future directions

It would be beneficial if future studies on the benefits of cherries for exercise recovery addressed the following outstanding issues:

a: Which type of cherry product is most effective in enhancing exercise recovery, juice from fresh‐frozen cherries, juice from concentrate, or some type of cherry extract? A study comparing different products is superior to comparing published studies for resolving this issue.

b: What is the optimal daily dose for products that have been shown to be effective? To date, the daily intake has replicated the early studies that showed some benefits and there have been no dose comparison exercise recovery studies.

c: What are the relative contributions to enhanced recovery of pre‐loading on the days prior to an exercise insult versus only loading on the day of and the days after the exercise? When is it too late to achieve a benefit?

d: What is the mechanism by which cherry juice enhances exercise recovery? Recovery effects are generally attributed to antioxidant and anti‐inflammatory effects, but enhanced functional recovery has been more consistently shown than systemic antioxidant or anti‐inflammatory effects.

e. Are other tart cherry cultivars as effective or more effective than Montmorency cherries in enhancing exercise recovery? To date, all studies have used products from Montmorency cherries.

## 8 PERSPECTIVES

This review of the literature on the use of cherry juice in exercise recovery highlights the importance of timing for optimizing the beneficial effects. The term precovery is used to emphasize this timing issue. It takes several days of consuming cherry juice to induce measurable changes in markers of antioxidant status^8^, ^31^ or systemic inflammation.^10^, ^33^ While the absence of such changes after a dosing regimen, prior to an exercise insult, does not preclude a subsequent post‐exercise benefit, it does point to the potential for a greater benefit with a precovery versus recovery regimen. This conclusion is supported by the fact that the only study failing to show a benefit of cherry juice for a measure of muscle function on the days after an exercise insult did not provide juice on the days prior to the exercise.^3^ Based on the extensive research on tart cherry juice, it can be recommended that consuming at least one serving a day for several days prior to an exercise insult will provide an accelerated recovery of function on the days after the exercise.

## Data Availability

This is a review paper with no data

## References

[1] Hill JA , Keane KM , Quinlan R , Howatson G . Tart cherry supplementation and recovery from strenuous exercise: a systematic review and meta‐analysis. Int J Sport Nutr Exerc Metab. 2021;31(2):154‐167. 33440334 10.1123/ijsnem.2020-0145

[2] Collins J , Maughan RJ , Gleeson M , et al. UEFA expert group statement on nutrition in elite football. current evidence to inform practical recommendations and guide future research. Br J Sports Med. 2021;55(8):416. 33097528 10.1136/bjsports-2019-101961

[3] Abbott W , Brashill C , Brett A , Clifford T . Tart cherry juice: no effect on muscle function loss or muscle soreness in professional soccer players after a match. Int J Sports Physiol Perform. 2020;15(2):249‐254. 31188696 10.1123/ijspp.2019-0221

[4] Jacob RA , Spinozzi GM , Simon VA , et al. Consumption of cherries lowers plasma urate in healthy women. J Nutr. 2003;133(6):1826‐1829. 12771324 10.1093/jn/133.6.1826

[5] Kelley DS , Rasooly R , Jacob RA , Kader AA , Mackey BE . Consumption of Bing sweet cherries lowers circulating concentrations of inflammation markers in healthy men and women. J Nutr. 2006;136(4):981‐986. 16549461 10.1093/jn/136.4.981

[6] Connolly DA , McHugh MP , Padilla‐Zakour OI . Efficacy of a tart cherry juice blend in preventing the symptoms of muscle damage. Br J Sports Med. 2006;40(8):679‐683. 16790484 10.1136/bjsm.2005.025429 PMC2579450

[7] Traustadóttir T , Davies SS , Stock AA , et al. Tart cherry juice decreases oxidative stress in healthy older men and women. J Nutr. 2009;139(10):1896‐1900. 19692530 10.3945/jn.109.111716 PMC3151016

[8] Howatson G , McHugh MP , Hill JA , et al. Influence of tart cherry juice on indices of recovery following marathon running. Scand J Med Sci Sports. 2010;20(6):843‐852. 19883392 10.1111/j.1600-0838.2009.01005.x

[9] Pigeon WR , Carr M , Gorman C , Perlis ML . Effects of a tart cherry juice beverage on the sleep of older adults with insomnia: a pilot study. J Med Food. 2010;13(3):579‐583. 20438325 10.1089/jmf.2009.0096 PMC3133468

[10] Schumacher HR , Pullman‐Mooar S , Gupta SR , et al. Randomized double‐blind crossover study of the efficacy of a tart cherry juice blend in treatment of osteoarthritis (OA) of the knee. Osteoarthritis Cartilage. 2013;21(8):1035‐1041. 23727631 10.1016/j.joca.2013.05.009

[11] Kelley DS , Adkins Y , Laugero KD . A Review of the Health Benefits of Cherries. Nutrients. 2018;10(3):368. 10.3390/nu10030368 PMC5872786 29562604

[12] Ducharme NG , Fortier LA , Kraus MS , et al. Effect of a tart cherry juice blend on exercise‐induced muscle damage in horses. Am J Vet Res. 2009;70(6):758‐763. 19496666 10.2460/ajvr.70.6.758

[13] Hillman AR , Taylor BCR , Thompkins D . The effects of tart cherry juice with whey protein on the signs and symptoms of exercise‐induced muscle damage following plyometric exercise. J Funct Foods. 2017;29:185‐192. doi:10.1016/j.jff.2016.12.026

[14] Bell PG , Stevenson E , Davison GW , Howatson G . The effects of montmorency tart cherry concentrate supplementation on recovery following prolonged, intermittent exercise. Nutrients. 2016;8(7):441. 10.3390/nu8070441 PMC4963917 27455316

[15] Bell PG , Walshe IH , Davison GW , Stevenson E , Howatson G . Montmorency cherries reduce the oxidative stress and inflammatory responses to repeated days high‐intensity stochastic cycling. Nutrients. 2014;6(2):829‐843. 24566440 10.3390/nu6020829 PMC3942735

[16] Bell PG , Walshe IH , Davison GW , Stevenson EJ , Howatson G . Recovery facilitation with Montmorency cherries following high‐intensity, metabolically challenging exercise. Appl Physiol Nutr Metab. 2015;40(4):414‐423. 25794236 10.1139/apnm-2014-0244

[17] Bowtell JL , Sumners DP , Dyer A , Fox P , Mileva KN . Montmorency cherry juice reduces muscle damage caused by intensive strength exercise. Med Sci Sports Exerc. 2011;43(8):1544‐1551. 21233776 10.1249/MSS.0b013e31820e5adc

[18] Brown MA , Stevenson EJ , Howatson G . Montmorency tart cherry (Prunus cerasus L.) supplementation accelerates recovery from exercise‐induced muscle damage in females. Eur J Sport Sci. 2019;19(1):95‐102. 30058460 10.1080/17461391.2018.1502360

[19] Kuehl KS , Perrier ET , Elliot DL , Chesnutt JC . Efficacy of tart cherry juice in reducing muscle pain during running: a randomized controlled trial. J Int Soc Sports Nutr. 2010;7:17. 20459662 10.1186/1550-2783-7-17 PMC2874510

[20] Lamb KL , Ranchordas MK , Johnson E , et al. No effect of tart cherry juice or pomegranate juice on recovery from exercise‐induced muscle damage in non‐resistance trained men. Nutrients. 2019;11(7):1593. 10.3390/nu11071593 PMC6683053 31337122

[21] McCormick R , Peeling P , Binnie M , Dawson B , Sim M . Effect of tart cherry juice on recovery and next day performance in well‐trained Water Polo players. J Int Soc Sports Nutr. 2016;13:41. 27895542 10.1186/s12970-016-0151-x PMC5109721

[22] Morehen JC , Clarke J , Batsford J , et al. Montmorency tart cherry juice does not reduce markers of muscle soreness, function and inflammation following professional male rugby League match‐play. Eur J Sport Sci. 2021;21(7):1003‐1012. doi:10.1080/17461391.2020.1797181 32672095

[23] Quinlan R , Hill JA . The efficacy of tart cherry juice in aiding recovery after intermittent exercise. Int J Sports Physiol Perform. 2019;15:368‐374. 10.1123/ijspp.2019-0101 31614329

[24] Wangdi JT , O'Leary MF , Kelly VG , Jackman SR , Tang JCY , Dutton J , Bowtell JL . Tart cherry supplement enhances skeletal muscle glutathione peroxidase expression and functional recovery after muscle damage. Med Sci Sports Exerc. 2021. doi:10.1249/MSS.0000000000002827. Epub ahead of print. PMID: 34772901. 34772901

[25] Levers K , Dalton R , Galvan E , et al. Effects of powdered Montmorency tart cherry supplementation on an acute bout of intense lower body strength exercise in resistance trained males. J Int Soc Sports Nutr. 2015;16(12):41. 10.1186/s12970-015-0102-y PMC4647629 26578852

[26] Levers K , Dalton R , Galvan E , et al. Effects of powdered Montmorency tart cherry supplementation on acute endurance exercise performance in aerobically trained individuals. J Int Soc Sports Nutr. 2016;26(13):22. 10.1186/s12970-016-0133-z PMC4880859 27231439

[27] Beals K , Allison KF , Darnell M , et al. The effects of a tart cherry beverage on reducing exercise‐induced muscle soreness. Isokinetics and Exercise Science. 2017;25(1):53‐63.

[28] Kupusarevic J , McShane K , Clifford T . Cherry gel supplementation does not attenuate subjective muscle soreness or alter wellbeing following a match in a team of professional rugby union players: a pilot study. Sports (Basel). 2019;7(4):84. 10.3390/sports7040084 PMC6524362 30959854

[29] Kirakosyan A , Seymour EM , Llanes D , Kaufman P , Bolling S . Chemical profile and antioxidant capacities of tart cherry products. Food Chem. 2009;115(1):20‐25.

[30] Ou B , Bosak KN , Brickner PR , Iezzoni DG , Seymour EM . Processed tart cherry products–comparative phytochemical content, in vitro antioxidant capacity and in vitro anti‐inflammatory activity. J Food Sci. 2012;77(5):H105‐H112. doi:10.1111/j.1750-3841.2012.02681.x 23163942

[31] Lynn A , Mathew S , Moore CT , et al. Effect of a tart cherry juice supplement on arterial stiffness and inflammation in healthy adults: a randomised controlled trial. Plant Foods Hum Nutr. 2014;69(2):122‐127. 24570273 10.1007/s11130-014-0409-x

[32] Wang H , Nair MG , Strasburg GM , Booren Am , Gray I , Dewitt Dl . Cyclooxygenase active bioflavonoids from Balaton tart cherry and their structure activity relationships. Phytomedicine. 2000;7(1):15‐19. 10782485 10.1016/S0944-7113(00)80016-1

[33] Bell PG , Gaze DC , Davison GW , George TW , Scotter MJ , Howatson G . Montmorency tart cherry (Prunus cerasus L.) concentrate lowers uric acid, independent of plasma cyanidin‐3‐O‐glucosiderutinoside. J Fun Foods. 2014;11:82‐90. doi:10.1016/j.jff.2014.09.004

[34] Bell PG , McHugh MP , Stevenson E , Howatson G . The role of cherries in exercise and health. Scand J Med Sci Sports. 2014;24(3):477‐490. 10.1111/sms.12085 23710994

[35] Kirakosyan A , Seymour EM , Wolforth J , et al. Tissue bioavailability of anthocyanins from whole tart cherry in healthy rats. Food Chem. 2015;171:26‐31. 25308638 10.1016/j.foodchem.2014.08.114

[36] Keane KM , Bell PG , Lodge JK , et al. Phytochemical uptake following human consumption of Montmorency tart cherry (L. Prunus cerasus) and influence of phenolic acids on vascular smooth muscle cells in vitro. Eur J Nutr. 2016;55(4):1695‐1705. 26163338 10.1007/s00394-015-0988-9

